# Widespread Cutaneous Indeterminate Dendritic Cell Tumor (IDCT) With ETV3::NOAC2 Rearrangement Successfully Treated With PUVA Therapy: A Case Report

**DOI:** 10.1155/crh/8013335

**Published:** 2024-11-29

**Authors:** Akshay Deshpande, Zbigniew Rudzki, Jaideep Bhat, Matthew Pugh, Susan Rose, Shankara Paneesha

**Affiliations:** ^1^Department of Haematology, University Hospitals Birmingham NHS Foundation Trust, Birmingham, UK; ^2^Department of Histopathology, University Hospitals Birmingham NHS Foundation Trust, Birmingham, UK; ^3^Department of Dermatology, University Hospitals Birmingham NHS Foundation Trust, Birmingham, UK; ^4^West Midlands Regional Genetics Laboratory, Birmingham Women's and Children's NHS Foundation Trust, Birmingham, UK; ^5^Institute of Cancer and Genomic Sciences, University of Birmingham, Birmingham, UK

## Abstract

The unique histiocytic entity of indeterminate dendritic cell tumor (IDCT) is known to cause diagnostic conundrum and treatment dilemmas with no published consensus on either. We report a rare case of cutaneous IDCT with ETV3::NOAC2 rearrangement providing further evidence to its association with this condition. With its ease of administration and minimal side effects, PUVA therapy can be successfully used to treat cutaneous forms of IDCT.

## 1. Introduction

Indeterminate dendritic cell tumor (IDCT) or indeterminate histiocytosis is an exceedingly rare form of histiocytosis. The first case was reported by Wood et al. in 1985 [1], and less than 100 cases have been reported in the last 4 decades [2]. IDCT has been classified under the “*L*” group as per the 2016 Histiocytic Society classification [2], the fifth edition of WHO classification of hematolymphoid tumor groups IDCT under Langerhan cells and other dendritic cell neoplasms [3]. The dendritic cells constituting these tumors have a similar morphological appearance to Langerhan cells with S100 and CD1a positivity but typically lack Birbeck granules and langerin (CD207) expression [2, 4]. More than 80% of the cases are limited to the skin [5] with no age or sex predilection reported [6]. There is limited understanding of the pathogenesis due to the rarity of the disease but associations with ETV3::NCOA2 rearrangement [2, 7] and BRAFV600E [8] mutation have been reported. A quarter of the cases have been associated with other hematological malignancies, most commonly CMML [5]. Clinical course and prognosis are not firmly established [9]. No standard of care exists with a variety of treatment options including skin electron beam therapy, ultraviolet A and B therapy, topical and systemic steroids, systemic chemotherapy, and BRAF/MEK inhibitors tried individually or in combination with varying success [2, 5].

We hereby present a case of widespread cutaneous IDCT with ETV3::NCOA2 rearrangement with evidence of clonal hematopoiesis who was successfully treated with psoralen plus ultraviolet A therapy.

## 2. Case Presentation

**Figure 1:**
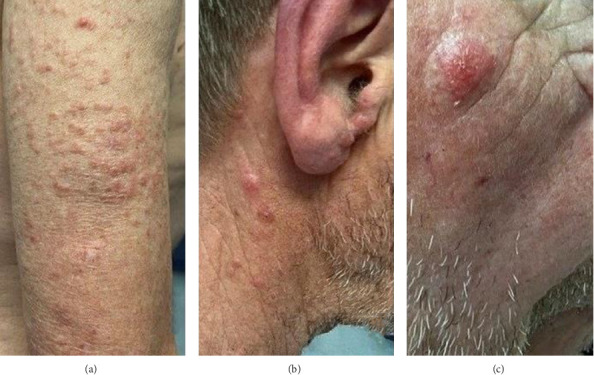
Nodular skin lesions at presentation (a) and rapidly spreading to involve the neck pinna and face (b, c).

A 76-year-old male presented with a 1-month history of itchy lumps around his right elbow (Figure 1(a)) and left flank. As there was a history of recent COVID booster vaccination, the initial thoughts went with pityriasis lichenoides or an unusual reaction to the vaccine. The lesions did not respond to topical steroids and progressed to involve up to 75% of the body surface including the face (Figures 1(b) and 1(c)). The patient did not have any systemic symptoms on presentation. On examination, he did not have any lymphadenopathy or hepatosplenomegaly. Full blood count and liver and renal function tests were normal.

**Figure 2:**
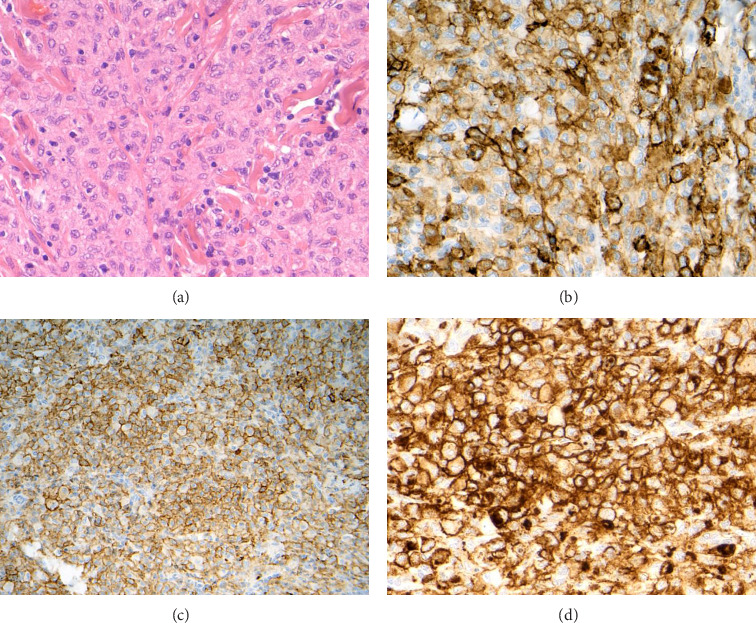
Neoplastic cells have histiocytoid morphology and do not show any striking atypia (H&E, objective 40x) (a). Expression of CD1a (objective 40x) (b), CD14 (objective 20x) (c), and CD56 (objective 40x) (d).

Two skin biopsies, an excision of a right thigh nodule and a punch from the left flank lesion showed identical changes. These were well-defined dermal nodules extending into the dermal–epidermal border without any signs of epidermotropism. The nodules comprised of large histiocytoid cells with ample eosinophilic cytoplasm and pale polymorphic nuclei and inconspicuous nuclear grooves (Figure 2(a)). The immunophenotype of these cells was CD1a+ (Figure 2(b)), Lysozyme+, Fascin+, CD14+ (Figure 2(c)), CD4+, CD163−, CD68−, S100+/−, Langerin−, F13A−, OCT2−, Cyclin D1−/+, ALK1−, CD21−, CD23−, CD2−, CD3−, CD5−, CD7−, CD8−, TCR Beta−, TCR Delta−, CD30−, CD15−, CD15−, MPO−, CD34−, CKIT−, TdT−, P53+/− (zonal, not strong), ki67 10%–25% (locally variable), CD43+, LCA+, CD123−/+, CD56+ (Figure 2(d)), and BRAF VE1−. EBV EBER was not expressed. Genetics was requested using the National Genomic Test Directory tests M117.1, M117.2, and M117.15 which encompass a range of changes found in histiocytosis. This resulted in the detection of the ETV3::NCOA2 rearrangement as the only abnormality.

All the above features were in keeping with the diagnosis of IDCT.

A staging CT scan and bone marrow trephine biopsy and aspiration did not show any abnormality apart from minimal erythroid hyperplasia. SNP and NGS array analyses of DNA extracted from the bone marrow aspirate detected loss of Y chromosome and biologically significant variant in ASXL1 (VAF 25%). Since the blood counts were normal and the bone marrow did not show any dysplastic features, this mutation was considered to represent clonal hematopoiesis of indeterminate potential (CHIP). Tests are currently ongoing to check for ASXL1 variant in the skin biopsy specimen.

As there is no consensus on the standard of care, the case was discussed in local and national MDT. After reviewing the available literature, it was decided to proceed with a trial of psolaren-plus ultraviolet A (PUVA) therapy. The patient received the same twice weekly, and all skin lesions had completely resolved in 8 weeks (16 treatments). The patient remains in complete remission 6 months post-treatment. Dermatology and Hematology teams are following him up closely.

## 3. Discussion

Dendritic cells are antigen-presenting cells, which regulate antigen-specific *T*-cell responses [10]. These cells along with monocytes/macrophages are derived from the same hematopoietic progenitor cells: colony-forming unit–monocyte (CFU-M) [6]. Monocytes have shown differentiation to dendritic cells in vitro [5], possibly explaining the association between myeloid neoplasms and IDCN. Indeterminate or immature dendritic cells are precursors to Langerhan cells and interstitial dendritic cells [6].

Brown et al. reported recurrent ETV3::NCOA2 rearrangement in three cases of IDCN suggesting a clonal etiology [7].^.^Nuclear receptor coactivator 2 (NCOA2) is a transcription factor involved in the coactivation of hormone receptors and adipose tissue homeostasis while Ets variant 3 (ETV3) inhibits Ras-dependent macrophage proliferation [5, 7]. NCOA2 translocations have been described in various cancers such as acute myeloid leukemia (fusion with KAT6A), acute lymphoblastic leukemia (fusion with ETV6), mesenchymal chondrosarcoma (fusion with HEY1), and prostate and colon cancer, whereas ETV3 alterations have been reported in brain and breast cancer [5, 7]. O'Malley et al. reported a case of IDCT associated with angioimmunoblastic T-cell lymphoma and evidence of BRAF-V600E [11], and Thurner et al. reported a case of IDCT with BRAF V600E mutation in long-term remission with MEK/BRAF inhibitors [12].

In a review by Horna et al. of the 79 reported cases, 22% (17) were associated with underlying hematological malignancy including six cases of chronic myelomonocytic leukemia (CMML), three cases of acute myeloid leukemia (AML), and four cases of follicular lymphoma [5]. Lie et al. have reported an association with low-grade B-cell neoplasms in 31% of the cases, but the patient numbers were small [13]. In our case, there was no coexisting diagnosis of myeloid malignancy or MDS but clonal hematopoeisis was detected supporting an association between myeloid malignancies and IDCN. Surprisingly, cases associated with myeloid neoplasms are limited to the skin as with this case [5].

The median age of diagnosis is 48 years with two case report of solitary lesions in newborns [5]. Horna et al. have reviewed 86 cases from 1985 to 2016 and found that 88% of the cases were limited to the skin. Multiple lesions are twice as common as compared to isolated lesions [5]. Classical presentation is in the form of papules and nodules sparing the palms and soles [5]. Leonine facies have been reported in two cases due to extensive facial involvement [8]. Lymph node involvement has been reported in isolation or along with cutaneous involvement especially in cases with known lymphoma. There have been isolated case reports of IDCN affecting the spleen, spine, conjunctiva, and pancreas [2]. In most cases, clinical course has been indolent [6].

The histological features and the immunophenotype of the present neoplasm unequivocally pointed out at an entity from the broad differential diagnosis circle of histiocytoses and related neoplasms. Langerhan cell histiocytosis was excluded on morphology (no obvious nuclear grooves and no eosinophils) and immunohistochemistry (negative langerin). Lack of emperipolesis and lack of OCT2 overexpression argued against Rosai–Dorfman disease. Known clinical data did not fit Erdheim–Chester disease. The degree of atypia fell short of a histiocytic sarcoma. The lesional cells did not appear blastic in contrast to what would be seen in a myeloid sarcoma with histiocytic/monocytic differentiation being unlikely. “Nonblastic” morphology and lack of TdT excluded plastic plasmacytoid dendritic cell tumor, which might have been initially considered due to the unusual expression of CD56. To our knowledge, this is the first case report of IDCN expressing CD56. More extensive S100 expression would be expected in an interdigitating dendritic cell sarcoma/tumor. Finally, the presence of the characteristic ETV3::NCOA2 rearrangement provided a positive and reassuring argument for IDCN.

A wide range of treatment options has been tried for cases of IDCT, and there have been reports of spontaneous remission as well. Localized excision, cryotherapy, and cauterization are possible treatment options for isolated skin lesions. As with our case, multiple reports show excellent responses to total electron beam therapy or phototherapy with PUVA or UVB, but relapses tend to be common needing repeat treatments and long-term follow-up data [13]. With an acceptable toxicity profile, ease of administration and excellent tolerability, we feel the use of PUVA as first-line therapy for widespread cutaneous IDCT. It has been reported that PUVA might not be effective for facial lesions and alternatives like low-dose methotrexate need to be considered [13]. Further data on the use of MEK inhibitors and immune modulators such as lenalidomide and thalidomide are needed. No death has directly been attributed to cutaneous IDCT.

Management of systemic IDCT and IDCT associated with myeloid malignancies and lymphomas seems to be more challenging. A wide range of treatments including CHOP/CVP-based regimens, combinations of vinca alkaloids, methotrexate, etoposide, steroids, and cytarabine have been reported [2, 13]. All these regimens seem to be bespoke and with no consensus. The outcomes in this group are less encouraging with relapse rates up to 50% and mortality rates of over 20% in a 4-year follow-up period [2].

## 4. Conclusion

The classical histology and absence of langerin along with clinical presentation should suffice for making a diagnosis of IDCT. ETV3::NCOA2 rearrangement can have diagnostic and disease-defining implications for IDCT, and further research in this regard is warranted. PUVA therapy shows excellent response with minimum side effects in cutaneous IDCT.

## Data Availability

For original data, please contact akshay.deshpande@nhs.net.
